# Strong and Deadly Futures: Co-Development of a Web-Based Wellbeing and Substance Use Prevention Program for Aboriginal and Torres Strait Islander and Non-Aboriginal Adolescents

**DOI:** 10.3390/ijerph18042176

**Published:** 2021-02-23

**Authors:** Mieke Snijder, Lexine Stapinski, James Ward, Briana Lees, Cath Chapman, Katrina Champion, Michael Doyle, Ian Watson, Rachael Sarra, Amanda Lear, Sophia Garlick Bock, Maree Teesson, Nicola Newton

**Affiliations:** 1 The Matilda Centre for Research in Mental Health and Substance Use, Sydney Medical School, Faculty of Health and Medicine, University of Sydney, Sydney 2006, Australia; m.snijder@ids.ac.uk (M.S.); briana.lees@sydney.edu.au (B.L.); cath.chapman@sydney.edu.au (C.C.); katrina.champion@sydney.edu.au (K.C.); sophia.garlickbock@sydney.edu.au (S.G.B.); maree.teesson@sydney.edu.au (M.T.); nicola.newton@sydney.edu.au (N.N.); 2 South Australia Health and Medical Research Institute, Adelaide 5000, Australia; james.ward@uq.edu.au; 3 Centre for Research Excellence in Indigenous Health and Alcohol, Discipline of Medicine, Central Clinical School, University of Sydney, Sydney 2050, Australia; michael.doyle@sydney.edu.au; 4 Gilimbaa, Indigenous Creative Agency, Brisbane 4101, Australia; ian@gilimbaa.com.au (I.W.); sarracreative@gmail.com (R.S.); amanda@amandalear.com.au (A.L.)

**Keywords:** Aboriginal and Torres Strait Islander, prevention, alcohol, cannabis, tobacco, substance use, school-based program, universal prevention, wellbeing, harm minimisation, Indigenous

## Abstract

School-based programs can effectively prevent substance use; however, systematic reviews and consultation with stakeholders identified a need for effective, culturally inclusive programs for Aboriginal and/or Torres Strait Islander (hereafter Aboriginal) youth. This paper describes the development of *Strong & Deadly Futures*, a six-lesson, curriculum-aligned wellbeing and substance use prevention program that was designed for, and with, the Aboriginal youth. Formative reviews and consultation recommended that the program (i) combine effective components of mainstream prevention with cultural elements, highlighting Aboriginal cultural strengths; (ii) avoid stigma and celebrates the cultural diversity by catering to both Aboriginal and non-Aboriginal students; and (iii) use digital technology to enhance engagement, implementation and scalability. Guided by an Appreciative Inquiry approach, the program was developed in partnership with an Indigenous Creative Design Agency, and four schools in New South Wales and Queensland, Australia. Aboriginal (*n* = 41) and non-Aboriginal students (*n* = 36) described their role models, positive aspects of their community and reasons to avoid substance use; these formed the basis of an illustrated story which conveyed the key learning outcomes. Feedback from teachers, students and content experts supported the acceptability of the program, which will be evaluated in a subsequent randomised controlled trial.

## 1. Introduction

Despite resilience and a continuous strong connection to culture, the ongoing impacts of colonisation, disempowerment and inequity have an intergenerational impact on the wellbeing of Aboriginal and Torres Strait Islander adolescents [1]. This intergenerational impact contributes to five-times higher levels of psychological distress, and on average, initiation of substance use two to six years earlier among Aboriginal and Torres Strait Islander compared to non-Aboriginal adolescents [2,3]. Earlier onset and escalation of substance use are risk factors for substance use disorders and associated problems later in life, such as poorer education outcomes and co-occurring mental health problems [4,5,6].

Prevention of adolescent substance use has been identified as a key strategy to improve Aboriginal and Torres Strait Islander Peoples’ wellbeing [7,8,9]. Schools are an important setting for substance use prevention as they offer the opportunity to reach the majority of adolescents and provide cohort benefits, in that students adopt new knowledge and beliefs as a group and these group norms can reduce alcohol, tobacco and cannabis use [10,11]. In mainstream populations, school-based substance use prevention programs have been shown to be effective in delaying and reducing substance use, improving knowledge, attitudes and changing intentions [12,13]. A recent review identified two Australian school-based programs, the School Health and Alcohol Harm Reduction Project (SHAHRP) [14] and *Climate Schools* [15], with good evidence of effectiveness in preventing substance use and related harms among Grade 8/9 students (approximately age 13 to 15) [16]. These prevention approaches have the potential to make a significant public health impact, as for every year that substance use initiation is delayed, the odds of developing a substance use disorder is reduced by 9% [17].

Although the benefits of school-based prevention have been established in mainstream populations, there is a lack of programs that have been shown to effectively prevent, delay and/or reduce substance use among Aboriginal and Torres Strait Islander adolescents [18,19]. Snijder et al. [19] identified four evaluations of Australian prevention programs reporting reductions in alcohol, cannabis or analgesic use among Aboriginal and Torres Strait Islander adolescents. Two of these programs were school-based, but the evaluations were published over two decades ago and methodological limitations of the studies precluded statistical tests of program efficacy [20,21]. The most recent evaluation of a school-based prevention program did not demonstrate overall improvements in substance outcomes for Aboriginal and Torres Strait Islander adolescents [22]. Notably, no program evaluations for Aboriginal and Torres Strait Islander adolescents were identified that made use of digital technology to support program delivery [19]. This is despite the potential advantages afforded by web-based or computerised programs, such as program fidelity, student engagement and the potential to overcome barriers to implementation among disadvantaged, hard to reach or culturally diverse populations [23].

We aim to address the gap in school-based substance use prevention programs that are effective for Aboriginal and Torres Strait Islander adolescents. This paper describes the formative research, program co-development and focus testing of a culturally inclusive school-based substance use prevention program co-developed with Aboriginal and Torres Strait Islander and non-Aboriginal students (aged 12–14 years), teachers and other stakeholders.

## 2. Materials, Methods and Results

**Figure 1:**
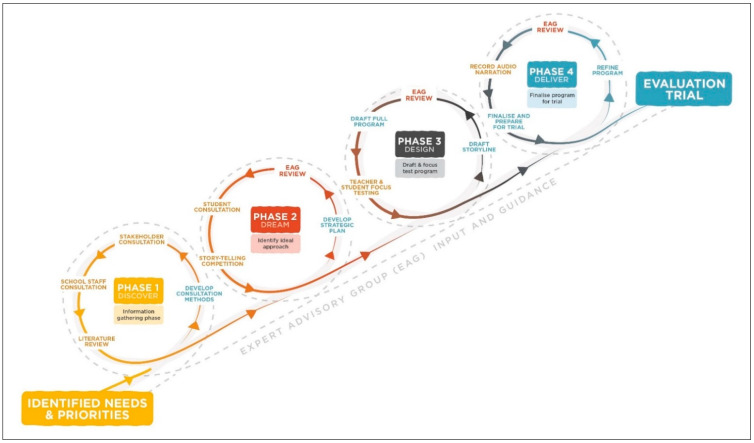
Development process guided by Appreciative Inquiry approach.

The scope, objectives and project methodology were developed through formative consultation with key stakeholders (school-based, service providers, government and academic) based in New South Wales, Queensland, Western Australia, Northern Territory and Victoria. The school-based program was co-developed through participatory research in partnership with teachers and students from four schools in New South Wales and Queensland (two urban independent schools, one regional and one remote state school), an Indigenous creative design agency (*Gilimbaa*) and an expert advisory group consisting of six Aboriginal and ten non-Aboriginal experts in school-based substance use prevention, and Aboriginal substance use and health research. Additionally, the research team received advice from representatives of the Departments of Education in Queensland and Northern Territory. The development process is illustrated in Figure 1.

The co-development process adopted a strength-based approach, which is recommended for Aboriginal and Torres Strait Islander health promotion as it has a potentially empowering effect on the social and emotional wellbeing [24]. Program development followed the Appreciative Inquiry model, an inclusive and affirmative approach that identifies and builds on existing strengths and achievements in the community [25]. Appreciative Inquiry consists of four phases, each of which is described in this paper:

1. **Discover**, identifying what is currently happening and what is working well;
2. **Dream**, identifying the ideal approach the target group would like to see implemented;
3. **Design**, taking the best of what is currently working and what the target group would ideally like to see happening and designing a program based on this; and
4. **Deliver**, developing action plans and implementing the program designed in Design phase.

### 2.1. Phase 1 (Discover): Information Gathering

#### 2.1.1. Stakeholder and School Staff Consultations

Consultations were undertaken with key stakeholders and school staff to (i) identify the current substance use prevention programs for Aboriginal and Torres Strait Islander youth; and (ii) identify the approach and key messages that work well and should be included in prevention programs for Aboriginal and Torres Strait Islander youth. Table 1 provides an overview of the participants, methods and topic guide for the consultations. Stakeholders were identified from key organisations (e.g., Aboriginal education, services, academic) and through expert advisory group contacts and networks. Stakeholders (*n* = 24) were recruited to the study via email invitation; of these, eight did not reply or were unable to commit time for consultation. Sixteen stakeholders participated in a phone interview conducted by MS, BL or IW. Where appropriate, stakeholders were invited to provide ongoing input into the project via membership of the expert advisory group. In addition, staff at four schools who had previously collaborated with the research team were invited to participate in a focus group. Any staff member with interest/involvement in health curriculum or Aboriginal and Torres Strait Islander education was invited to participate via a central contact person at the school. A single focus group was facilitated by MS and IW in each of the four schools. School staff were given the option of consultation via individual interview if they were unable or preferred not to attend the focus group.

Notes were taken during stakeholder consultations and focus groups were audio recorded. A summary of the findings was sent to all stakeholders and schools to ensure participants agreed with the main outcomes.

##### Findings: Current Prevention Resources for Aboriginal and Torres Strait Islander Youth

Stakeholders and school staff were not aware of any substance use prevention programs specifically designed for Aboriginal and Torres Strait Islander adolescents. Consultees identified a need for reliable resources that are easily accessible for community members, school staff (teachers, school counsellors and nurses) and parents. School staff reported using a variety of prevention approaches: (i) guest speakers (e.g., ex-users, health workers); (ii) resources based on the Australian or state-based curriculum, such as PowerPoints and workbooks; (iii) *Family Wellbeing* empowerment program with at-risk girls [26]; (iv) pastoral care diaries for students to reflect in; and (v) the *Climate Schools* alcohol and cannabis prevention program [15,27,28]. Outside the school context, common prevention approaches included vocational courses that target disadvantaged students (including Aboriginal and Torres Strait Islander students) and engagement centres specifically for students who are not attending school.

School staff noted that current approaches seem to work for some students, but that substance use continues to be a challenge in the community and novel approaches are needed. School staff identified that successful elements of prevention programs were interactive sessions, personal stories from people who have used substances, empowering messages and providing scenarios that are relatable to students’ lives. In line with effective substance use prevention principles [29,30], school staff did not consider lessons solely focused on information provision and long-term consequences of substance use to be effective, as adolescents are generally less concerned about future consequences.

##### Findings: Culturally Inclusive Prevention Materials

During the formative consultations, stakeholders and school staff voiced a strong preference and need for a program that is culturally appropriate and meets the needs of the whole classroom, which often includes both Aboriginal and Torres Strait Islander and non-Aboriginal students. School staff and other stakeholders noted that Aboriginal and Torres Strait Islander students often have friends from diverse cultural backgrounds and face similar issues as their non-Aboriginal peers:

‘*The reality is that Aboriginal and non-Aboriginal students mix inside and outside of school. A program should reflect this dynamic*’.

(Aboriginal service provider and academic)

‘*More important is that programs are based on reality, the students are hanging out with each other, there is not really any separation between Aboriginal and non-Aboriginal students in terms of their friend groups and [they] are going through life’s issues like dealing with drugs and alcohol together. Programs need to reflect this*’.

(Aboriginal education officer)

Consultees also emphasised current programs do not reflect this diversity. It was thought that providing programs uniquely to Aboriginal and Torres Strait Islander students who attend schools with non-Aboriginal students could be received as stigmatising:

‘*Separating Indigenous and non-Indigenous kids within drug and alcohol programs can be really tricky. While there may be some very deep cultural issues to consider, … there is also a danger of making it seem like only Aboriginal young people are vulnerable to drugs and alcohol, which is not the case. Sometimes, Aboriginal students feel they are being targeted in schools*’.

(Aboriginal service provider)

‘*There is definitely risk of stigmatisation when you take Indigenous students out of the classroom, especially when it is to discuss alcohol and other drug issues’*.

(non-Aboriginal academic)

There was, however, recognition of the unique experiences of Aboriginal and Torres Strait Islander students, including different pressures and expectations around substance use, experiences of racism, grief and loss and distinct protective factors such as cultural identity, pride and community:

‘*Indigenous-specific content is important because reasons for using substances and the ways of healing from substance abuse/addiction are different*’.

(Aboriginal government-based stakeholder)

‘*Grief and loss are important topics to cover with Indigenous students—it is a big problem that lends itself to alcohol and drug abuse—need to provide other ways of thinking than just “alcohol/drugs are the only way to deal with my loss”*’.

(Aboriginal community health service provider)

In view of these considerations, most consultees preferred a program for mixed-cultural classrooms, that celebrated Aboriginal and Torres Strait Islander cultural strengths and diversity and provided an opportunity for Aboriginal and Torres Strait Islander students to discuss unique experiences separately from non-Aboriginal students.

‘*[It] should be integrated and one program to present to all students—teach all students about the different reasons for using substances, risk factors, ways of healing from addiction from different cultural perspectives (Caucasian, Indigenous etc.,)—this way all students learn more about their own culture, and others. Another way we can encourage reconciliation*’.

(Aboriginal government-based stakeholder)

‘*Rather than separating the programs it should be a general statement, but with the option for Aboriginal students to have a separate section, because there might be certain issues that the Aboriginal students would like to discuss separately from the non-Aboriginal students*’.

(Aboriginal academic)

Table 2 outlines the elements of effective prevention identified by stakeholders and school staff. Overall, consultees voiced strong support for a program that is appropriate and inclusive for both Aboriginal and Torres Strait Islander and non-Aboriginal students. It was recommended that the program take a harm minimisation approach and empower youth by building self-efficacy, resilience, coping and decision-making skills. In terms of substance use content, consultees suggested that prevention should focus on alcohol, tobacco and cannabis as these are the most commonly used substances among Aboriginal and Torres Strait Islander and non-Aboriginal adolescents. It was recommended that key messages include the short-term consequences of use, focus on relationship-level factors by exploring the impact of social influence and peer pressure, and communicate that substance use can affect everyone. School staff and other stakeholders agreed that there should be a focus on pride in cultural identity among both Aboriginal and Torres Strait Islander and non-Aboriginal students. Finally, the format should be interactive. Teachers highly recommend the use of technology to align with the students’ preferences and integrate flexibility around accessibility for various skill levels.

#### 2.1.2. Systematic Literature Review

To complement the recommendations derived from these formative consultations, we reviewed the academic and grey literature to determine the modifiable risk and protective factors for substance use among Aboriginal and Torres Strait Islander people, and the effective substance use prevention components identified internationally for Indigenous youth. A full description of the methods, search terms and findings of these systematic reviews are provided in the published review protocols [31,32] and results [19,33]. Table 3 summarises the implications for effective program development arising from these systematic reviews, and reviews of mainstream substance use prevention literature [16,29].

#### 2.1.3. Discover Phase: Conclusions

Taking together, findings from our consultation and literature review in the information gathering phase indicated four key considerations in developing a culturally appropriate school-based prevention approach for Aboriginal and Torres Strait Islander adolescents:

1. Adopt a comprehensive prevention strategy (beyond information provision) that is empowering, incorporates skills training and explores the impact of social influence and psychological distress on substance use;
2. Combine core effective elements of mainstream programs with cultural elements, given the target factors for Aboriginal and Torres Strait Islander and non-Aboriginal adolescents are well aligned [33], and international evidence suggests that mainstream programs that are adapted to align with cultural identity and practices are effective in preventing substance-related harms among Indigenous adolescents [19];
3. Avoid stigma by developing a program for both Aboriginal and Torres Strait Islander and non-Aboriginal students that is inclusive, empowering and celebrates the cultural diversity within Australian classrooms;
4. Align with the Australian schools’ curriculum and make use of technology for program delivery to capitalise on advantages of this delivery method, including student engagement, scalability and ease of implementation.

### 2.2. Phase 2 (Dream): Identifying the Ideal Approach

#### 2.2.1. Strategic Plan for Program Development

Drawing on the four key considerations for program development derived from the information gathering phase, a strategic plan for program development was developed in consultation with the Expert Advisory Group. Given the finding that effective programs typically combine core elements of mainstream programs with cultural elements, the *Climate Schools* substance use prevention programs were identified as candidates for cultural adaptation to suit a diverse classroom of Aboriginal and Torres Strait Islander and non-Aboriginal youth. *Climate Schools* is based on effective principles and program elements identified in the mainstream substance use prevention literature (see Table 3). It is the only Australian school-based prevention program with strong evidence for effectiveness [16], and has been evaluated in seven randomised controlled trials [28,34,35,36,37,38,39,40]. Compared to usual health education, the *Climate Schools* programs have been found to reduce the frequency of students’ alcohol and cannabis use and truancy for up to two years following the intervention [15,37]. The interactive programs use illustrated storytelling delivered via an online platform to communicate substance use prevention messages, reinforced by interactive classroom activities facilitated by the teacher. The theoretical foundations of *Climate Schools* are the social influence and harm minimisation approaches to substance use prevention. Harm minimisation refers to strategies aimed at reducing substance use as well as reducing harms when substance use does occur [27,41,42]. The social influence model [11,43] incorporates three components within a harm minimisation approach: (i) Providing developmentally appropriate and credible information about alcohol and other substances that are directly relevant to the students; (ii) providing realistic information about substance use norms among adolescents, including dispelling myths and inflated perceptions of the extent that peers use substances; (iii) resistance-skill training, which encourages students to identify sources of pressure to use substances and learn skills to manage these situations.

In addition to its effectiveness and strong theoretical foundation, *Climate Schools* was thought to be well-suited for adaptation. First, because the storytelling used in *Climate Schools* aligns well with Aboriginal and Torres Strait Islander ways of sharing knowledge [44,45]. Second, the core prevention messages are delivered via stories about, and dialogue between, a group of adolescents, providing peer to peer delivery of the core content. Third, the program incorporates many of the desirable and effective components identified through our information gathering stage (outlined in Table 3). It incorporates skills training and substance use knowledge acquisition which were identified as effective components of prevention for Indigenous youth internationally [19], and addresses factors identified in the risk and protective factors review, including truancy, peer use and ability to resist peer pressure and normalisation of substance use [33]. Fourth, the web-based delivery of *Climate Schools* provides an interactive approach that aligns with teacher and student preferences to use digital technology to enhance learning [46]. Furthermore, web-based delivery provides flexibility around tailoring to various skill levels as the learning pace can be adjusted and audio narration can be integrated to accommodate lower literacy levels. A final advantage was that development of the culturally adapted program could draw on expertise within our team from the developers of the original *Climate Schools* programs (MT, NN).

A final plan for cultural adaptation of the *Climate Schools* program was presented to the expert advisory group for approval, who indicated strong support for the proposal [47]. In brief, program adaptation aimed to combine the effective prevention components of *Climate Schools*, while incorporating Aboriginal and Torres Strait Islander cultural elements. Consistent with *Climate Schools*, the target age group for the adapted program was Grade 8 students (approximately age 13). However, in view of evidence that in some communities Aboriginal and Torres Strait Islander adolescents initiate substance use at a younger age than the general population [2], the program was designed with the flexibility to also accommodate younger students (Grade 7). Like the *Climate Schools* program, the adapted program was grounded in a social influence and harm minimisation approach, with key messages delivered peer to peer via an illustrated story about a group of adolescents.

#### 2.2.2. Consultation with Adolescents

Prioritising the perspectives of the target group, Aboriginal and Torres Strait Islander adolescents, was a central consideration for program development. Identifying the core stories and elements to include in the program was achieved in collaboration with students in four partner schools. Students in these schools were selected to participate from Aboriginal and Torres Strait Islander leadership programs (*n* = 47) and in the fourth school all Grade 7 and 8 students participated (*n* = 30). Written parental consent was obtained for student participation and audio/video recording of the sessions. Students were not reimbursed for the sessions as they were completed within school hours as part of their drug education curriculum. The sessions were structured to be interactive and enjoyable to maximise student engagement. In the ‘Dream’ phase, student perspectives and views about the important messages for substance use prevention were gathered via photovoice focus groups and creative sessions. Photovoice is a research method in which data were collected via photography and analysed through group discussions [18]. Students from the four schools were provided with a digital camera and asked to take photos over a two-week period. Following this, the students participated in small group discussions where they shared their photos before discussing these with the larger group. Subsequent focus groups were held with the students to discuss their knowledge, priorities, educational and support needs relating to substance use. Table 4 provides an overview of the participants, methods and topic guides for the session. Following the focus groups, students participated in a creative session which involved poster-making (using their photos) or role-playing relevant scenarios. The sessions were audio and video recorded.

To seek broader input beyond these schools, a national storytelling competition for Aboriginal and Torres Strait Islander adolescents was organised. Adolescents (aged 12–14) were recruited to the competition via advertising on social media and information sent out to schools and Aboriginal and Torres Strait Islander community-controlled health services around the country. Participants in the storytelling competition were asked to submit a short written, visual or audio piece to capture stories related to the same topics examined in the student consultations (see Table 4).

A detailed description of the qualitative findings of the photovoice project and storytelling competition will be in a forthcoming paper. Table 5 summarises the findings from these student consultations that informed the key messages and content of the prevention program.

### 2.3. Phase 3 (Design): Co-Design of the Strong & Deadly Futures Program

#### 2.3.1. Co-Design of the Illustrated Story, Learning Summaries and Classroom Activities

The program was designed to cover six lessons, each involving: (i) an episode of a web-based illustrated story; (ii) student and teacher summaries; and (iii) interactive classroom activities. All learning outcomes and content were mapped to the Australian National Health and Physical Education Curriculum and NSW Personal Development, Health and Physical Education by an expert consultant teacher. Elements of each lesson were designed to be hosted on a web-based platform (https://strongdeadly.org.au/), accessible by teachers and students. The prevention program was designed for Year 8 students with flexibility to accommodate implementation in Year 7 according to school structure (e.g., combined Year 7–8 classes in some schools), or local context. Prevention is most effective when implemented prior to initiation of substance use [48], and this flexibility was incorporated given evidence of earlier substance use initiation compared to the general population in some Aboriginal and Torres Strait Islander communities [2]. Key learning outcomes and skill development for each lesson were based on existing evidence regarding effective components of prevention (as summarised in Table 1 and Table 2), gathered during the consultations and literature reviews conducted in Phase 1.

Using the stories shared by the students in Phase 2, the research and *Gilimbaa* team iteratively developed the story arc and characters. The overall story arc was presented to, and approved by, the Expert Advisory Group. Following this approval, scenes were drafted around key learning outcomes, content and scenarios emerging from the student consultations and literature reviews. Script drafting and story illustration for each lesson were facilitated by a working group at *Gilimbaa*, which consisted of five experienced young Aboriginal communication professionals, artists and designers from Goreng Goreng, Larrakia, South Seas and Quandamooka Nations. The working group provided input on content, language, illustrations and cultural sensitivity. The research team explicitly ensured the inclusion of substance use prevention content based on research evidence. Draft scripts and illustrations were approved by the Expert Advisory Group.

The resulting illustrated story follows a core group of five secondary school students (two Aboriginal and/or Torres Strait Islander and three non-Aboriginal students) as they face challenges relating to psychological stress and substance use. Peripheral characters who feature in the story include siblings, cousins and parents of the central characters, elders and health workers. Table 6 shows an overview of the key learning outcomes and scenes in each lesson.

Teacher and student summaries were developed to provide information on the key learning outcomes of each lesson and the relevant evidence-base. The *Gilimbaa* working group reviewed all summaries to ensure the appropriateness for the target audience. Classroom activities were designed to further solidify knowledge and strategies learned in the illustrated story. These activities were based on previous *Climate Schools* activities [15] and other programs for Aboriginal and Torres Strait Islander people [26] or specifically developed in consultation with the Aboriginal working group and the expert advisory group to highlight cultural strengths.

#### 2.3.2. Co-Development of Program Name and Logo

The name of the program was generated by the *Gilimbaa* working group. The working group proposed a variety of names with a strength-based, positive and inclusive connotation. The names were informally tested with Aboriginal and/or Torres Strait Islander Year 7–10 students in a mentoring program who voiced strong support for *Strong & Deadly Futures*. The name was selected to ensure that the program is inclusive for all students when delivered in a classroom. ‘Strong’ highlights the strength-based approach of the program, ‘Deadly’ is a commonly used word in Aboriginal and Torres Strait Islander communities and means cool or awesome, and ‘Futures’ indicates how this program will develop a positive foundation in students’ lives.

**Figure 2:**
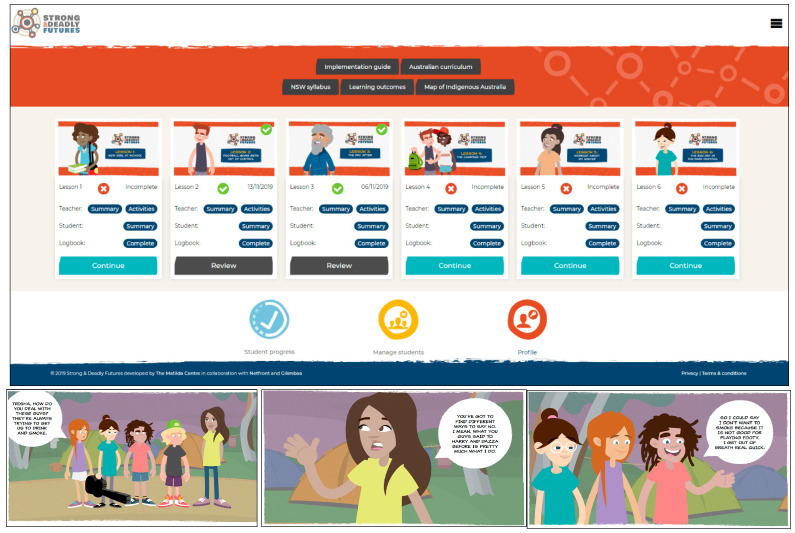
Program logo, example scenes and online platform design for *Strong & Deadly Futures*.

The logo and web design (Figure 2) were illustrated by Jenna Lee (a Larrakia, Wardaman and Karajarri artist from *Gilimbaa*). The logo represents a complex individual surrounded by educational and social support, with multiple possible strong and deadly futures before them as they are informed and feel empowered following the program.

#### 2.3.3. Student and Teacher Focus Testing of Draft Program Materials

Following the development of the draft lesson materials and the online portal, all preliminary content was focus tested with teachers and students to (i) gain feedback on the overall storyline, scripts and illustrations, and (ii) inform revisions to language, content and illustrations.

##### Participants and Procedures

Focus groups were conducted with eight teachers and 48 students from the four partner schools in NSW and QLD who were involved during Phase 2. Teachers were presented with an overview and content of *Strong & Deadly Futures* and feedback was elicited on: (i) the usability of the program and accompanying website, (ii) the characters and example storyboards and (iii) the degree to which they believed their students would engage with, and relate to, the characters, language, relationships, storylines and content.

Students viewed the illustrations of each character and participated in the creative sessions where they acted out scripts from two lessons in small groups. Facilitators led a discussion, using prompt questions to gain feedback on the acceptability of the content, messages, illustrations, language and characters (appearance, authenticity, perceived age). Testing also included exploration of students’ engagement with the characters and storylines, as well as recognition of key messages. All consultations were recorded with permission from the participants.

##### Data Analysis

General inductive analysis [49] was conducted to identify themes from the focus testing data. Coding was done independently by MS and IW (from *Gilimbaa*). Themes identified by each reviewer were compared and retained based on consensus. Themes were: (i) relatability of the key messages and content; (ii) overall feedback on illustrations; (iii) potential for student engagement; (iv) strengths of web-based delivery; (v) language use; (vi) classroom activities; (vii) useability; (viii) suitability for regional areas; (ix) the idea of illustrated stories as a way to convey key messages; and (x) recalling the key messages.

##### Focus Testing Results: Teacher Feedback

There were several points of feedback informing refinements to the program. These related to specific storylines and content, language and usability of the lesson materials, as outlined below.

Usability of the Program. *Strong & Deadly Futures* was perceived as highly usable by all teachers because of the web-based delivery, links to the curriculum and the large variety of activities. The web-based delivery was perceived positively because it makes program materials easily accessible in a central place and reduces preparation time for the teachers: ‘*teachers are time poor so having resources already written up, so we don’t have to do it, is great, I don’t have to spend hours beforehand preparing lessons*’. This was especially important in non-metropolitan schools where ‘*teachers often teach out of their expert area*’, as it will ensure the students get all the information they need, irrespective of the teacher’s expertise. Web-based delivery was considered beneficial as it gives teachers a way to track where the students are up to: ‘*seeing what the kids have done is handy*’. The importance of the program’s alignment with the Australian Curriculum was mentioned in each focus group and contributed to the increased program usability perceived by the teachers: ‘*it’s important that it links with the curriculum’; ‘perfect fit with [personal development health physical education] PDHPE curriculum’*; ‘*I’d love to pop this into our Year 7 plan*’. Teachers also identified the alternative uses for the program, such as using it in mentoring or homework groups or using in a ‘*topical way when students are being affected by [issues relating to substance use], this can be used with small groups with selected students*’. Usability was further increased by the variety of activities provided. Teachers noted that this meant they can easily adapt the program to students with different ability levels: ‘*it will serve for different levels of ability as well*’; ‘*there is a massive range of students at the school, separate activities and differentiate within the activities*’. Information in the teacher summary documents was sufficient: ‘*teacher summary looks like enough information’*; ‘*not too much not too little’*. Content specifically for Aboriginal and Torres Strait Islander students were found to be informative: ‘*notes for Indigenous students look good’*.

Design of Illustrated Story and Characters. The concept of using illustrated stories was well received in all focus groups. Teachers noted that the illustrations would be appropriate to use with Year 7 and 8 students ‘*because it is less confronting than having real life people in it, especially for the age group*’. The use of illustrated stories in a substance use prevention program was perceived as innovative (‘*students will probably like it as it is very different from other things that they have done before*’), relatable (‘*it’s good that the program is with kids and new scenarios, not something that has been developed 20 years ago, it has stories they will be able to relate to*’) and engaging for students (‘*takes away from just reading paper scenarios*’). Especially in remote and regional schools it was received positively: ‘*previous stuff has been created in urban areas, so it is nice to have something that fits in with regional and remote*’. Teachers viewed the illustration designs positively: ‘*illustrations look good and the students will interact with them*’. The characters in the stories were perceived as realistic and relatable with a good balance between male and female characters and no stereotypical characters: ‘[I] *can imagine kids that we have that look like this*’; ‘*I reckon they’ll relate to them*’.

Content of Illustrated Stories. The stories were perceived to be easy to read, relatable, accurate and answered questions students might have about the topics discussed, especially in the regional and remote schools:

‘*a lot of them will have experienced going to a new school*’;

‘*The information in the cartoon is answering the questions that the students might get while reading it, like what is a standard drink, what effects does alcohol have, what should I do if my family or friends are offering it, what do I do when my friends are intoxicated, benefits of not drinking, showing it is cool to not drink*’;

‘*Students will experience effects of peer pressure at home, with family*’;

‘*This is good because our students are exposed to [substance use] a lot*’.

The teachers were also positive about the messages that were integrated into the storylines, including that the negative aspects of drinking are portrayed (e.g., ‘*vomit on his shirt*’) and that the friends also disapproved of the depicted drinking behaviour: ‘*it is also nice that the judging about Dazza taking it too far also comes from his friends*’.

Student Engagement. In all focus groups teachers agreed that the web-based delivery would increase student engagement with the content as it aligns with the students’ interests, allows them to work individually and at their own pace: ‘*students love doing things individually and using ICT*’; ‘*Interactive resources are good, they don’t like being talked at, and sitting at a computer with a headphone and listening to videos*’. The integration of audio in addition to written text was thought likely to enhance the engagement ‘*It is good that there will be voice overs, otherwise the students will complain about having to read too much*’. Finally, teachers agreed that the variety of activities available for students will keep them engaged: ‘*changing the activities is good to keep students engaged*’; ‘*different things for the students to do, not slide after slide, it will keep them engaged*’.

##### Focus Testing Results: Student Feedback

Student feedback was focussed on acceptability of the story and scenarios, appropriateness of the learning outcomes and the illustrations, characters and language.

Acceptability of Content. All students agreed that the overall storyline and most individual scenarios were realistic and reflected relatable situations, including moving school, attending football games, going on camping trips and having crushes on classmates. Overall, the dialogue, interactions and scenarios were found to be realistic:

‘*The peer pressure situation between Billy and his cousin works well, more than if it was his brother*’.

‘*When I moved school I sort of felt the same way, I moved a couple of times from place to place*’.

‘*When I moved school I used to keep on calling my old friends*’.

‘*…we have conversations with friends when we’re worried about our friend*’.

However, some interactions received mixed responses, for example in one scenario where the main character discusses her worries with her mother, some students responded that they would discuss worries with their parents, whereas other students would not. The students who did not feel comfortable discussing issues with their parents, indicated they would not really talk to anyone about their worries. Related to another scenario, some (male) students indicated that they would not directly talk to an older sister about their substance use: ‘*I would maybe talk about this with my brother, but not my sister*’.

Appropriateness and Delivery of Learning Outcomes. In a group discussion, the students were able to accurately identify the key messages that were being communicated in each lesson. They provided feedback on both the content of the key messages and the way these messages were delivered. Overall, key messages were well received by the students and found to be realistic and relatable. In terms of decision-making skills, students agreed that discussing pros and cons were an important skill for adolescents to learn: ‘*we don’t always look at all the sides of a decision*’. Students were positive about the alcohol-related content, including standard drinks information (‘*standard drinks info explains it well*’), reasons not to drink (‘*it makes sense that if you want to achieve things to suggest not really drinking’*) and alternative activities to drinking. Students made suggestions for changes to further increase engagement, such as explaining standard drinks in a visual way and to promote drinking in moderation rather than abstinence: ‘*change to not drink too much*’.

In terms of tobacco use, refusal strategies and reasons provided for not smoking were perceived to be appropriate, realistic and relatable. The following reasons were considered the most convincing and important for students: smoking being unpleasant (‘*everyone always says “you stink”’*), bad for health (‘*can kill people*’, ‘*gives you cancer’*) and expensive (‘*cost lots of money’*). Students were positive about messages related to talking with friends and adults about substance-related issues: ‘*good idea to talk to an adult about drugs and alcohol when having issues*’; ‘*Something important to talk about with your friends*’. Finally, in relation to how messages were shared, students were positive about the peer to peer nature of the program and that the adolescent characters shared key messages, rather than the adults: ‘*the fact that it is coming from the kids is making it better’*. Students made suggestions for changes where this was not yet the case: ‘*It doesn’t sound good to have the character say “my mum says”, change that*’.

Characters, Illustrations and Language. Across all focus groups, students found the characters to be relatable, engaging and were interested in exploring their stories and interactions. The five core characters were perceived to be realistic and reflective of 12-year-olds ‘*they’re not all the same height, which is realistic*’; ‘*they look different ages*’. One of the Aboriginal core characters was particularly well received: ‘*Joe embraces his Aboriginal culture, with his Aboriginal socks on and beads in his hair*’. One of the non-Aboriginal core characters was received as looking too old and mother-like. Students suggested changes to her hair and clothing. In terms of the peripheral (older) characters, students commented that ‘*they all look the part of what they do and are like’*: The ex-footy player turned health worker ‘*looks like someone who could work in community services*’, the youth worker ‘*looks like a youth worker for an Indigenous town*’ and the Aboriginal Elder ‘*looks wise and like an Aboriginal Elder*’. The students were very positive about the inclusion of a dog in the story.

The students were positive about the illustrations; they thought that the colour scheme and fonts, illustration backgrounds and story settings (e.g., campground, youth centre, house) were appropriate. Students highlighted some issues, and provided suggestions, regarding some incongruent facial expressions.

Students expressed mixed responses to ‘Aboriginal English’ words, such as yarn, mob and deadly. Some students were comfortable using these words, while others mentioned that it is mainly adults that use those words. Students also had mixed responses about substance-related terms. For example, some students suggested using the word ‘weed’ when referring to cannabis, whereas other students would not use this term, but rather use ‘yarndi’ or refer to smoking cannabis as just ‘smoking’. In terms of alcohol, some students suggested using the word ‘grog’ and talk about being ‘tipsy’ rather than being ‘drunk’, however, the majority of students were comfortable with ‘drunk’ and ‘alcohol’.

### 2.4. Phase 4 (Deliver): Finalising the Program and Preparing for Implementation

#### 2.4.1. Refining the Program in Response to Student and Teacher Feedback

During the final phase of the program development, changes were implemented to refine the illustrations, narrative, lesson summaries and classroom activities in response to teacher and student feedback. Supplementary Table S1 outlines the major refinements made in response to each item of feedback.

#### 2.4.2. Expert Review of Final Program

Once refinements to the program based on teacher and student feedback had been made, the expert advisory group were invited to conduct a final review of the program to ensure all content was (i) aligned with the evidence and best practice, (ii) culturally sensitive and appropriate, and (iii) the functionality of the online platform was acceptable. Surveys were sent to 25 members of the expert advisory group, of whom 14 completed the survey (4 identified as Aboriginal people). Each expert was asked to review one of the six lessons, which were allocated based on their expertise. After reviewing the illustrated story, classroom activities and student and teacher summaries, the experts completed a survey assessing cultural appropriateness, relevance, depth and usefulness of the lessons’ content, as well as the functionality of the online platform. Questions were answered using a 5-point Likert scale ranging from ‘not at all’ to ‘very’. Each section had the option to provide additional comments in an open text field.

**Figure 3:**
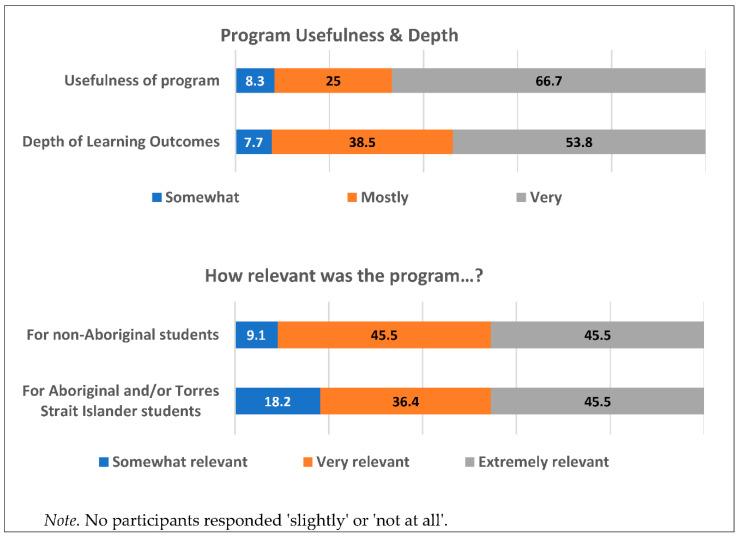
Expert advisory group feedback on final program.

Overall, there was positive feedback from the experts on the different elements of the program. Figure 3 summarises the feedback from the experts on questions about the overall program. Experts thought that the substance use content was relevant for Year 7 and 8 students: ‘*the content is extremely relevant and the advantage of this type of activity is it allows flexibility*’; ‘*the visual presentation of the consequences of cannabis use was nice*’. Feedback indicated the learning outcomes of each lesson were covered in sufficient depth for Year 7 and 8 students: ‘*it was detailed in addressing its aims whilst keeping the students engaged*’. The program was thought to be relatable to both Aboriginal and/or Torres Strait Islander as well as non-Aboriginal students:

‘*The context is very engaging and it is easy to use the website. It is great to have a diversity of people and to have both dark and fair Aboriginal characters*’.

‘*I think the cartoon does a great job at catering for students with low literacy levels*’.

‘*I think the cartoon does a great job at being relevant and inclusive for Aboriginal and Torres Strait Islander students*’.

Final refinements were made to the program in line with feedback from expert review, as summarised in Supplementary Table S2.

#### 2.4.3. Finalising the Program and Preparing for Implementation

The final stage of development involved finalising the materials and program website in preparation for evaluation. Although web-based delivery is an advantageous feature of the program, PDF and video files of the illustrated story, student and teacher materials were created so that the program could also be accessed offline, via a USB device. Offline access to program materials circumvents accessibility issues that may arise in schools with poor or intermittent Internet connections. In addition, audio narration of the illustrated story was implemented (for both web-based and online versions) to increase student engagement and ensure the program is inclusive and accessible for students with a range of abilities and literacy levels. Voice-overs for the core adolescent characters were narrated by five Aboriginal and/or Torres Strait Islander and three non-Aboriginal students who were involved in the earlier phases of program development. The supporting characters were narrated by a member of the research team, *Gilimbaa* team members (David Williams, Wakka Wakka and Gilimbaa Director and Co-founder; Jill Robinson, Bundjalung) and special guests (including Roxanne McDonald, an Aboriginal actress from the Mandandanjii and Darambal tribes; Joe Williams, a Wiradjuri suicide prevention advocate; and Corey Bobongie, a young actor from the Gurang Guran and Birri tribes from South Seas Islands descent). A video summary of the program and co-development process was also developed, and is available at https://strongdeadly.org.au/.

The final component of the Deliver phase is implementation and evaluation of the program to assess its effectiveness in preventing substance use and related harms among Aboriginal and/or Torres Strait Islander and non-Aboriginal Year 7 and 8 students. A pilot trial has been completed and will be published in a forthcoming paper. Funding has been secured for a national, cluster randomised controlled trial to test the efficacy of the program in 24 schools [31]. To ensure that wellbeing priorities and diversity between communities is reflected, additional consultation will be conducted prior to the trial with Aboriginal and/or Torres Strait Islander adults and adolescents in each of the 24 communities serviced by the participating schools. A local Aboriginal or Torres Strait Islander facilitator will be employed to lead these consultations, and local adaptations will be made to the program to reflect community feedback (e.g., edits to the language, illustrations and cultural elements/activities). Full details about the trial and pre-trial consultation phase can be found in the study protocol [32].

## 3. Discussion

This paper described the formative research, co-development and focus testing of an Australian-first web-based school-based wellbeing and substance use prevention program that is culturally inclusive and designed for implementation with both Aboriginal and Torres Strait Islander and non-Aboriginal students aged 12 to 14. This program was developed to address the need for evidence-based prevention to reduce the disproportionately higher rates of substance-related harms experienced by Aboriginal and Torres Strait Islander adolescents, compared to non-Aboriginal adolescents [18,19].

This formative research highlighted important considerations when developing substance use prevention for Aboriginal and/or Torres Strait Islander youth, which included adopting a comprehensive prevention approach, combining effective prevention elements with cultural elements, avoiding stigma and using digital technology to enhance engagement and accessibility. The importance of providing a comprehensive prevention approach and combining effective prevention elements with cultural elements have been discussed in detail in previous research [9,18,19,29].

The multicultural school environment presents a challenge for ensuring the cultural appropriateness and relevance of drug and alcohol education, and avoiding stigma or marginalisation [19]. Aboriginal and Torres Strait Islander youth have a unique historical and cultural background, but mostly attend school with non-Aboriginal peers [48]. Our formative consultations confirmed the importance of avoiding stigma and promoting inclusivity, and thus a culturally inclusive prevention program was developed that conveys that substance use is an issue that affects people from all cultural backgrounds. Incorporating traditional Aboriginal and Torres Strait Islander ways of sharing knowledge through the use of storytelling was a format that appealed to both Aboriginal and/or Torres Strait Islander and non-Aboriginal youth, and also enabled the core prevention messages to be conveyed peer-to-peer. Embedding Aboriginal and Torres Strait Islander perspectives, practices and knowledge into teaching can have numerous benefits for students, including improved engagement, school-connectedness and learning [49,50]. Teachers in our study were enthusiastic to implement a program that highlights Aboriginal and Torres Strait Islander cultural strengths and saw the benefits of this approach for Aboriginal and Torres Strait Islander and non-Aboriginal students. The Australian curriculum now mandates that Aboriginal and Torres Strait Islander cultures, knowledge and histories are embedded in Australian schooling, however there remain ongoing concerns about tokenism, and many teachers report that lack of resources and confidence are a barrier to incorporating cultural perspectives into teaching [49,51]. This study demonstrated the value and appeal of a ready-made, school-based wellbeing and substance use prevention program that incorporates and celebrates Aboriginal and Torres Strait Islander cultures. It is critical that education and school policies ensure teachers are supported to implement empowering, decolonising curriculum and wellbeing practices such as these that provide Aboriginal and Torres Strait Islander students with meaning opportunities to achieve their full potential.

Our formative research contributes to emerging evidence that using digital technology in the delivery of prevention and health promotion programs is well received and has benefits for marginalised youth, including Aboriginal and/or Torres Strait Islander youth [23,52]. Benefits of web-based prevention includes the ability to overcome issues with accessibility and financial barriers for disadvantaged populations as well as high implementation fidelity and reduced costs and personnel requirements associated with delivery [23,53]. Accessibility of *Strong & Deadly Futures* was perceived to be a strength as the web-based delivery allows for the easy integration of text, images and audio to cater for different literacy levels of students. This formative research further indicated widespread support for web-based delivery as the familiar, interactive and digital format likely increases adolescents’ engagement with the program. It reduces teachers’ preparation time and provides standardisation of taught content, which was found to be especially important in rural areas where teachers regularly teach out of their expertise area. Systematic reviews have repeatedly shown that computer- or Internet-delivered prevention programs are more effective than education as usual in reducing the uptake and delaying the age of onset for substance use among adolescents in mainstream (i.e., non-Aboriginal specific) populations [16,53,54,55,56,57]. The upcoming cluster randomised controlled trial of *Strong & Deadly Futures* will confirm whether the observed benefits identified in our formative research and previous studies, translate into measurable benefits for Aboriginal and Torres Strait Islander and non-Aboriginal students [58].

## 4. Strengths and Limitations

One limitation of this research was that consultations were only conducted with four schools in east coast states of Australia (NSW and QLD). Aboriginal and Torres Strait Islander peoples are not a homogenous group and consist of many cultures and communities. Therefore, information provided in the consultations does not necessarily reflect experiences of Aboriginal and/or Torres Strait Islander youth in other parts of Australia. To supplement the perspectives from youth in these four schools, the *Gilimbaa* working group was composed of young designers and communication experts from Goreng Goreng, Larrakia, South Seas and Quandamooka Nations. This group provided feedback on the stories to avoid locally specific cultural elements and ensure common cultural elements across different Aboriginal and Torres Strait Islander nations were highlighted (e.g., the importance of Elders, connection to Country) [59]. The nationwide storytelling competition also aimed to address this limitation, although the majority of entries were still from NSW, Queensland, Victoria, South Australia and Tasmania. Prior to implementing *Strong & Deadly Futures* in additional schools during the randomised controlled trial, community consultations will be undertaken to tailor *Strong & Deadly Futures* to the local context where needed.

## 5. Conclusions

The next step for this research is a cluster randomised controlled trial to assess the efficacy of *Strong & Deadly Futures* in improving social and emotional wellbeing and empowerment, and reducing substance-related harms amongst Aboriginal and/or Torres Strait Islander and non-Aboriginal adolescents [58]. This will be the first trial of a web-based school-based substance use prevention program for Aboriginal and/or Torres Strait Islander adolescents. It will provide knowledge to address this critical evidence gap in culturally inclusive substance use prevention, and if efficacious, *Strong & Deadly Futures* provides a sustainable and scalable alternative to standard drug education that can be implemented widely to improve the wellbeing of young Aboriginal and Torres Strait Islander and non-Aboriginal Australians.

## Figures and Tables

**Table 1:** Participants and methods of the information gathering consultations.

	Participants	Methods	Topic Guide
Stakeholder	*n* = 16 (56% Aboriginal) Service providers: *n* = 5 School-based: *n* = 6 Government: *n* = 1 Academic: *n* = 4	Structured phone interview	(i) What current prevention materials are used with Aboriginal and Torres Strait Islander students? (ii) Should prevention programs be integrated or separate for Aboriginal and Torres Strait Islander and non-Aboriginal youth? (iii) What messages should be included in prevention materials for Aboriginal and Torres Strait Islander students?
School staff	*n* = 26 (31% Aboriginal) 4 schools: School 1 (Public rural NSW) *n* = 5 School 2 (Public regional NSW) *n* = 6 School 3 (Private rural QLD) *n* = 14 School 4 (Private urban QLD) *n* = 1	Focus Group/interview	(i) What are the current approaches school staff are using for substance use prevention with Aboriginal and Torres Strait Islander students? (ii) Are there differences in approaches between Aboriginal and Torres Strait Islander and non-Aboriginal students? (iii) What would the optimal prevention approach be? (iv) What prevention messages should be included?

**Table 2:** Consultation findings: Elements to include for effective of substance use prevention among Aboriginal and Torres Strait Islander adolescents.

Recommended Elements	Example Quote from Stakeholder/School Staff Consultation
Content to address misconceptions about substance use, alternatives to using and where to get help.	‘*Important to focus on help seeking behaviours, highlight to people where they can find help. And ask the students where they go to find help as* *well*’. (Aboriginal academic and former service provider)
2. Empowering messages and skill development, e.g., decision-making and refusal skills.	‘*Taking an empowerment approach is likely to be beneficial, because this can address the underlying life stressors and provide the students with strategies to cope withthis*’. (Aboriginal service provider and academic)
3. Positive content, including positive role models and success stories of people who do not use substances.	‘*Appropriate adult role modelling for positive change*’. (Aboriginal education officer)
4. Dealing with peer pressure.	‘*Messages should focus on peer and group effects*’. (Aboriginal education officer)
5. Engaging modules.	‘*Modules should be interactive and include videos to keep engagement*’. (Aboriginal community health service provider)
6. Interactive approaches with a mix of activities.	‘*Doing enjoyable activities that builds their capacity and skills and broadens their experiences*’. (Aboriginal academic and former service provider)
7. Use of technology.	‘*Today, so many kids are tech-focused, would be good for the program to be computer or app-based. This would be more appropriate than teacher facilitating the* *modules*’. (Non-Aboriginal academic)

**Table 3:** Evidence-based program elements and target risk and protective factors that the ideal substance use prevention approach for Aboriginal and Torres Strait Islander youth should include.

Program Elements	Target Risk and Protective Factors ^4^
*Elements identified in review of international programs for Indigenous adolescents ^1^*: Substance use knowledge enhancement Skill development Developed with Aboriginal and Torres Strait Islander community Cultural knowledge enhancement *Elements identified in review of mainstream substance use prevention literature ^2,3^*: Harm minimisation approach Social influence model Implemented early before use starts Developmentally appropriate resources Peer leadership Interactive approach	*Individual:* Higher level of general education (P) Greater knowledge about substances and their effects (P) Higher psychological distress (R) Use of one substance increases risk for other substance use (R) Boredom (R) *Relationship:* Positive family and community role models (P) Supportive family and peer environment (P) Peer/family substance use (R) Peer pressure to use substances (R) *Cultural:* Engagement with and connection to Aboriginal and Torres Strait Islander culture (P) Normalisation of substance use (R)

**Sources**: ^1^ Snijder, Stapinski [19]; ^2^ Newton, Deady [29]; ^3^ Lee, Cameron [16] ^4^ Snijder, Lees [33]. Key: P = Protective factors; R = Risk factors.

**Table 4:** Participants and methods in co-development of the program.

Consultation Stage	Participants	Methods	Topic Guide
Students	*n* = 77 (53% Aboriginal and/or Torres Strait Islander; 12–14 years) Per schools: School 1 (Public rural NSW) *n* = 30 School 2 (Public regional NSW) *n* = 12 School 3 (Catholic urban QLD) *n* = 15 School 4 (Catholic urban QLD) *n* = 20	Photovoice Focus group; Creative session (poster making or role playing)	Students completed a *photovoice* task capturing scenes related to: (i) Their role models; (ii) Positive reasons why people do not use drugs and alcohol; (iii) Positive interactions with friends; (iv) Things they love about their community. *Focus group:* (i) What do you learn at school about alcohol and other drugs? (ii) How does this fit with your experience of alcohol and other drugs in the real world? (iii) Are there other things you want to or think you should learn about in relation to alcohol and other drugs? (iv) Are there things you think your friends or siblings need to learn about alcohol and other drugs? (v) Can you tell us about a person or an experience that helped you to learn something important about alcohol or other drugs? *Creative session:* (i) Saying ‘no’ to something you don’t want to do; (ii) Helping a friend your concerned about; (iii) Asking for help; (iv) Activities that don’t involve alcohol or drugs.
Storytelling Competition	*n* = 13 (100% Aboriginal and/or Torres Strait Islander; 12–14 years)	Online Storytelling Competition	Participants submitted a short story or visual/audio piece, capturing scenes related to: (i) Their role models; (ii) Positive reasons why people do not use drugs and alcohol; (iii) Positive interactions with friends; (iv) Things they love about their community.

**Table 5:** Summary of student consultation and storytelling competition findings.

**Role Models**	**Positive Social Interactions**
Parents Teachers and other school staff Youth workers Grandparents Aunties/uncles Friends Classmates Famous sports players (football players, netball players) Book characters	Going to the movies Watching YouTube Playing sports Hanging out Doing arts and crafts Playing card games Going bushwalking Spending time with family Playing with pets Playing computer games
**Positive reasons not to use AOD**	**Things students love about their community**
Looking after animals: dogs, cats, horses, cows, parrots Costs money Impacts on playing sports (incl. hockey, netball, football, beach volleyball, soccer, racing, surfing, body boarding, frisbee) Friends and family: younger family members (need to be a role model for them and look after them), friends are not smoking, parents are not smoking, parents might disapprove (spending parents’ money). Playing computer games or musical instruments Studying, getting a good job and graduating Reduces self-esteem Reduces energy Prefer to be camping: out in nature, campfires	Lake, beach, river and swimming pool Youth centre/community centre Space and freedom to have fun Nature Shopping centre Playgrounds Aboriginal and Torres Strait Islander community connections

**Table 6:** Overview of the learning outcomes, targeted risk and protective factors and story elements included from the student consultations for each lesson of the prevention program.

Lesson	Module Learning Outcomes	Targeted Modifiable Factors	Story Elements from Consultations
1: New Girl at School	*Target: Psychological distress* Contributors to stress Strategies to cope with feelings of stress Decision making strategies Different ways to build self-efficacy	↓ Psychological distress ↑ Strategies to help cope with psychological distress ↑ build self-efficacy	Scene setting at basketball court at school Weighing up pros and cons when having to make difficult decisions Talking with mother Being new to school
2: Football Game Gets Out of Control	*Substance: Alcohol* Alcohol and the law Standard drinks and drinking guidelines Short- and long-term consequences of alcohol use Drink driving and its risks Harm minimisation strategies for self and others	↑ Alcohol consequences knowledge ↑ Positive role models	Scene setting at a football game Football player as role model Sports and looking after pets as important reason not to drink alcohol What students want to become when they grow up Car crash after drink driving
3: The Day After	*Substance: Alcohol* Challenging normative perceptions about alcohol use Reasons why young people do or do not drink alcohol Finding accurate information about substances online Reiterating harm minimisation strategies	↑ Alcohol consequences knowledge ↑ Harm minimisation knowledge ↓ Peer pressure ↓ Perceived peer substance use	Scene setting in youth/community centre Characters playing computer games and playing guitar Reasons to not smoke: friends are not smoking, smells bad, expensive
4: The Camping Trip	*Substance: Alcohol and tobacco* Health consequences of smoking Reasons why young people do or do not smoke Drug and alcohol refusal strategies Alternative activities to smoking and drinking The importance of Country and culture for the wellbeing of Aboriginal and Torres Strait Islander people	↑ Tobacco consequences knowledge ↑ Connection to culture ↑ Positive role models ↑ Alternatives to substance use ↓ Peer pressure ↓ Perceived peer substance use	Scene setting during a camping trip and around campfire Reasons to not smoke: hard to play sports, not part of culture Mother, football player, youth worker and elder (aunty) as role models
5: Worried About my Sister	*Substance: Cannabis* Helping someone going through a challenging time How to avoid other people’s substance use Consequences of cannabis use Cannabis and the law Dependence on cannabis	↑ Supportive network ↑ Cannabis knowledge ↑ Connection to culture	Scene setting at body of water and movie night at youth centre Sister who wants to be a lawyer Language used when friends are supporting each other
6: The ‘Big Day in the Park’ Festival	*Substance: Alcohol, tobacco and cannabis* Strategies to cope with feelings of stress Drug and alcohol refusal skills Helping someone going through a challenging time Alternative activities to smoking and drinking alcohol	↑ Supportive network ↑ Recreational alternatives to substance use ↓ Peer pressure	Scene setting at drug and alcohol-free community event Scene setting of alcohol consumption in older peers Football player as role model

↑ = promote, ↓ = reduce.

## Data Availability

The data presented in this study are not publicly available due to ethics approval requirements.

## Supplementary Materials

The following are available online at https://www.mdpi.com/1660-4601/18/4/2176/s1, Table S1: Teacher and student feedback and changes made to the program. Table S2: Expert advisory group feedback and changes made to the program.

## References

[1] Australian Institute of Health and Welfare (2019). Children Living in Households with Members of the Stolen Generations.

[2] Australian Institute of Health and Welfare (2006). Drug Use among Aboriginal and Torres Strait Islander Peoples: An Assessment of Data Sources.

[3] Azzopardi P.S., Sawyer S.M., Carlin J.B., Degenhardt L., Brown N., Brown A.D., Patton G.C. (2018). Health and wellbeing of Indigenous adolescents in Australia: A systematic synthesis of population data. Lancet.

[4] Degenhardt L., Stockings E., Patton G., Hall W.D., Lynskey M. (2016). The increasing global health priority of substance use in young people. Lancet Psychiat..

[5] Behrendt S., Wittchen H.U., Höfler M., Lieb R., Beesdo K. (2009). Transitions from first substance use to substance use disorders in adolescence: Is early onset associated with a rapid escalation?. Drug Alcohol Depend..

[6] Windle M., Spear L.P., Fuligni A.J., Angold A., Brown J.D., Pine D., Smith G.T., Giedd J., Dahl R.E. (2008). Transitions into underage and problem drinking: Developmental processes and mechanisms between 10 and 15 years of age. Pediatrics.

[7] King M., Smith A., Gracey M. (2009). Indigenous health part 2: The underlying causes of the health gap. Lancet.

[8] Australian Government (2013). National Aboriginal and Torres Strait Islander Health Plan 2013–2023 Canberra.

[9] Dickerson D., Baldwin J.A., Belcourt A., Belone L., Gittelsohn J., Kaholokula J.K.A., Lowe J., Patten C.A., Wallerstein N. (2018). Encompassing Cultural Contexts Within Scientific Research Methodologies in the Development of Health Promotion Interventions. Prev. Sci..

[10] Teesson M., Newton N.C., Barrett E.L. (2012). Australian school-based prevention programs for alcohol and other drugs: A systematic review. Drug Alcohol Rev..

[11] Botvin G.J. (2000). Preventing drug abuse in schools: Social and competence enhancement approaches targeting individual-level etiologic factors. Addict. Behav..

[12] Teesson M., Newton N.C., Slade T., Carragher N., Barrett E.L., Champion K.E., Kelly E.V., Nair N.K., Stapinski L.A., Conrod P.J. (2017). Combined universal and selective prevention for adolescent alcohol use: A cluster randomized controlled trial. Psychol. Med..

[13] Foxcroft D.R., Tsertsvadze A. (2012). Universal alcohol misuse prevention programmes for children and adolescents: Cochrane systematic reviews. Perspect. Public Health.

[14] McBride N., Farringdon F., Midford R., Meuleners L., Phillips M. (2004). Harm minimization in school drug education: Final results of the School Health and Alcohol Harm Reduction Project (SHAHRP). Addiction.

[15] Newton N.C., Teesson M., Vogl L.E., Andrews G. (2010). Internet-based prevention for alcohol and cannabis use: Final results of the Climate Schools course. Addiction.

[16] Lee N.K., Cameron J., Battams S., Roche A. (2016). What works in school-based alcohol education: A systematic review. Health Educ. J..

[17] Grant B.F., Stinson F.S., Harford T.C. (2001). Age at onset of alcohol use and DSM-IV alcohol abuse and dependence: A 12-year follow-up. J. Subst. Abuse.

[18] Lee K., Jagtenberg M., Ellis C., Conigrave K. (2013). Pressing need for more evidence to guide efforts to address substance use among young Indigenous Australians. Health Promot. J. Austr..

[19] Snijder M., Stapinski L., Lees B., Ward J., Conrod P., Mushquash C.J., Belone L., Champion K., Chapman C., Teesson M. (2020). Preventing substance use among Indigenous adolescents in the United States of America, Canada, Australia and New Zealand: A systematic review of the literature. Prev. Sci..

[20] Gray D., Sputore B., Walker J. (1998). Evaluation of an Aboriginal Health Promotion Program: A Case study from Karalundi. Health Promot. J. Austr..

[21] Sheehan M., Schonfeld C., Hindson E., Ballard R. (1995). Alcohol Education in an Indigenous Community School in Queensland, Australia. Drugs Educ. Prev. Polic..

[22] Malseed C., Nelson A., Ware R. (2014). Evaluation of a school-based health education program for urban Indigenous young people in Australia. Health.

[23] Chou W.Y.S., Prestin A., Lyons C., Wen K.Y. (2013). Web 2.0 for Health Promotion: Reviewing the Current Evidence. Am. J. Public Health.

[24] Fogarty W., Lovell M., Langenberg J., Heron M.J. (2018). Deficit Discourse and Strengths-based Approaches: Changing the Narrative of Aboriginal and Torres Strait Islander Health and Wellbeing.

[25] Murphy L., Kordyl P., Thorne M. (2004). Appreciative inquiry: A method for measuring the impact of a project on the well-being of an Indigenous community. Health Promot. J. Austr..

[26] Tsey K., Whiteside M., Elkins H.M., Bainbridge R., James C.Y., Wilson A. (2010). Empowerment and Indigenous Australian health: A synthesis of findings from family wellbeing formative research. Health Soc. Care Community.

[27] Newton N.C., Vogl L.E., Teesson M., Andrews G. (2009). Climate Schools: Alcohol Module: Cross-Validation of a School-Based Prevention Programme for Alcohol Misuse. Aust. N. Zeal. J. Psychiatry.

[28] Teesson M., Newton N.C., Slade T., Chapman C., Allsop S., Hides L., McBride N., Mewton L., Tonks Z., Birrell L. (2014). The CLIMATE schools combined study: A cluster randomised controlled trial of a universal Internet-based prevention program for youth substance misuse, depression and anxiety. BMC Psychiatry.

[29] Newton N., Deady M., Teesson T., Rosen P.B.A.A. (2014). Alcohol and Substance Use Prevention and Early Intervention. Early Intervention in Psychiatry: EI of Nearly Everything for Better Mental Health.

[30] United Nations Office on Drugs and Crime (2018). International Standards on Drug Use Prevention.

[31] Snijder M., Lees B., Ward J., Stearne A.E., Newton N.C., Stapinski L. (2019). Developing an ecological framework of factors associated with substance use and related harms among Aboriginal and Torres Strait Islander people: Protocol for a systematic review. BMJ Open..

[32] Snijder M., Stapinski L., Lees B., Newton N., Champion K., Chapman C., Ward J., Teesson M. (2018). Substance use prevention programs for indigenous adolescents in the United States of America, Canada, Australia and New Zealand: Protocol for a systematic review. J. Med. Internet Res..

[33] Snijder M., Lees B., Stearne A., Ward J., Garlick Bock S., Newton N., Ward J., Teesson M. (2021). An ecological model of drug and alcohol use and related harms among Aboriginal and Torres Strait Islander Australians: A systematic review of the literature. Prev. Med. Rep..

[34] Champion K.E., Newton N.C., Stapinski L., Slade T., Barrett E.L., Teesson M. (2016). A cross-validation trial of an Internet-based prevention program for alcohol and cannabis: Preliminary results from a cluster randomised controlled trial. Aust. N. Zeal. J. Psychiatry.

[35] Newton N.C., Andrews G., Champion K.E., Teesson M. (2014). Universal Internet-based prevention for alcohol and cannabis use reduces truancy, psychological distress and moral disengagement: A cluster randomised controlled trial. Prev. Med..

[36] Teesson M., Newton N.C., Slade T., Chapman C., Birrell L., Mewton L., Mather M., Hides L., McBride N., Allsop S. (2020). Combined prevention for substance use, depression, and anxiety in adolescence: A cluster-randomised controlled trial of a digital online intervention. Lancet Digit. Health.

[37] Newton N.C., Andrews G., Teesson M., Vogl L.E. (2009). Delivering prevention for alcohol and cannabis using the internet: A cluster randomised controlled trial. Prev. Med..

[38] Champion K.E., Newton N.C., Stapinski L., Teesson M. (2016). Effectiveness of a universal Internet-based prevention program for ecstasy and new psychoactive substances: A cluster randomised controlled trial. Addiction.

[39] Newton N.C., Teesson M., Mather M., Champion K.E., Barrett E.L., Stapinski L., Carragher N., Kelly E., Conrod P.J., Slade T. (2018). Universal cannabis outcomes from the Climate and Preventure (CAP) study: A cluster randomised controlled trial. Subst. Abuse Treat. Prev. Policy.

[40] Vogl L.E., Newton N.C., Champion K.E., Teesson M. (2014). A universal harm-minimisation approach to preventing psychostimulant and cannabis use in adolescents: A cluster randomised controlled trial. Subst. Abuse Treat. Prev. Policy..

[41] Toumbourou J.W., Stockwell T., Neighbors C., Marlatt G.A., Sturge J., Rehm J. (2007). Interventions to reduce harm associated with adolescent substance use. Lancet.

[42] Australia Co. (2017). National Drug Strategy 2017–2026.

[43] Botvin G.J., Griffin K.W. (2016). Prevention of substance abuse. APA Handbook of Clinical Psychology: Applications and Methods, Volume 3. APA Handbooks in Psychology^®^.

[44] Bessarab D., Ng’andu B. (2010). Yarning About Yarning as a Legitimate Method in Indigenous Research. Int. J. Crit. Indig. Stud..

[45] Geia L.K., Hayes B., Usher K. (2013). Yarning/Aboriginal storytelling: Towards an understanding of an Indigenous perspective and its implications for research practice. Contemp. Nurse.

[46] Lazard A.J., Pikowski J., Horrell L., Ross J.C., Noar S.M., Sutfin E.L. (2020). Adolescents’ and Young Adults’ Aesthetics and Functionality Preferences for Online Tobacco Education. J. Cancer Educ..

[47] Snijder M., Stapinski L., Lees B., Newton N., Champion K., Champan C. (2017). Positive Choices for young Aboriginal and Torres Strait Islander people Summary Report and Strategic Plan. Drug Strategy Branch.

[48] Ministerial Council for Education ECDaYA (2010). Aboriginal and Torres Strait Islander Education Action Plan 2010–2014.

[49] Baynes R. (2016). Teachers’ attitudes to including indigenous knowledges in the Australian science curriculum. Aust. J. Indig. Educ..

[50] Hansen J.J. (2016). Examining the Effectiveness of Including Aboriginal Perspectives to Engage Aboriginal Students in High School Science.

[51] Bishop M., Vass G., Thompson K. (2019). Decolonising schooling practices through relationality and reciprocity: Embedding local Aboriginal perspectives in the classroom. Pedagog. Cult. Soc..

[52] Brusse C., Gardner K., McAullay D., Dowden M. (2014). Social media and mobile apps for health promotion in Australian indigenous populations: Scoping review. J. Med. Internet Res..

[53] Champion K.E., Newton N.C., Barrett E.L., Teesson M. (2013). A systematic review of school-based alcohol and other drug prevention programs facilitated by computers or the internet. Drug Alcohol Rev..

[54] Rodriguez D.M., Teesson M., Newton N.C. (2014). A systematic review of computerised serious educational games about alcohol and other drugs for adolescents. Drug Alcohol Rev..

[55] Park E., Drake E. (2015). Systematic review: Internet-based program for youth smoking prevention and cessation. J. Nurs. Scholarsh..

[56] Wood S.K., Eckley L., Hughes K., Hardcastle K.A., Bellis M.A., Schrooten J., Demetrovics Z., Voorham L. (2014). Computer-based programmes for the prevention and management of illicit recreational drug use: A systematic review. Addict. Behav..

[57] Tait R.J., Spijkerman R., Riper H. (2013). Internet and computer based interventions for cannabis use: A meta-analysis. Drug Alcohol Depend..

[58] Stapinski L., Newton N., Snijder M., Doyle M., Champion K., Chapman C. (2018). APP1163416: Strong and Deadly Futures: A Cluster Randomised Controlled Trial of a Computerised Schoolbased Alcohol and Drug Prevention Program for Aboriginal and Torres Strait Islander Students.

[59] Salmon M., Doery K., Dance P., Chapman R., Gilbert R., Williams R., Raymond L. (2018). Defining the Indefinable: Descriptors of Aboriginal and Torres Strait Islander Peoples’ Cultures and their Links to Health and Wellbeing.

