# Supplementary material for: Strong and Deadly Futures: Co-Development of a Web-Based Wellbeing and Substance Use Prevention Program for Aboriginal and Torres Strait Islander and Non-Aboriginal Adolescents
Source: Int J Environ Res Public Health. 2021 Feb 23;18(4):2176. doi: 10.3390/ijerph18042176 (PMC7926400; doi:10.3390/ijerph18042176)
Supplement: Supplementary file 1 [file ijerph-18-02176-s001.pdf]

Table S1. Teacher and student feedback and changes made to the program.

Student feedback	Teacher feedback	Changes made
	Program usability	
	"Add in how long the activities will take to help teachers plan their lessons" "show which activities are must-do, compared to recommended activities"	Added in duration of each activity (based on students trying out the activity). Added in 'recommended' versus 'suggested' activities
	Storylines and content	
"I don't see a lot of support from mum […] maybe putting an arm round here" "Amy wouldn't tell that much to her mum, she wouldn't name names" "Mum could give her advice instead of just walking through the pros and cons"	"Mum needs to be more empathetic, and provide more support herself. Referring to a school counsellor straight away would not be something students would relate to." "simplify pros and cons, conversation goes on for too long"	Taken out mum referring her daughter to a counsellor, but offering herself for support. Drawn arm from mum around daughter. Taken out names of the characters and shortened conversation Shortened pros and cons discussing and included some advice from mum
Some students reported they would not discuss issues or substance use with a parent or older sister. Others reported that they would discuss these issues with family.		These scenes were left unchanged, given mixed views were voiced by the students. Furthermore, it was considered important to encourage help-seeking by providing this example of discussing problems with family as a normative teenage behaviour.
"Conversation between Amy and Tahlia is pretty long, there's no reason for Amy to say 'I don't have any friends here' or be so negative, it seems like it's been a pretty good day"	Can probably shorten some of the conversations, e.g. conversation with Tahlia	Reduced conversation between Amy (new girl at school) and Tahlia (friend from old school)
Billy's joke about where Amy lives goes too far "why would you make fun of where someone used to live?" Joking about something being from the 70s is too long ago	Kids probably don't know much about the 70 probably better to mention the 90s	Taken out bullying scene about where Amy used to live. Changed 70s to 90s.
Following the car crash, the youth worker needs to be more sympathetic and worried		Added in more sympathetic questions from youth worker to the youth.
	Messages	
Information about the standard drinks is maybe too long, too complicated and not really interesting/bit boring. Maybe visually represent this information, rather than in words.		Information about standard drinks presented on a visual flyer along with characters describing the information
Not drinking at all might be too much and not realistic (not good message). Drinking in moderation, not drinking every single day, get wasted every day. Related with sport it's OK to suggest not drinking at all when you're playing.		More strongly embedded the message around not drinking in a sports context
Youth worker messaging around finding reliable information online is too factual, needs to be more engaging		Taken out information online from the cartoon and moved into classroom activity.

	Illustrations and characters	
Inappropriate facial expressions:<br>"Characters are looking too happy in some scenes"<br>"Weird that sometimes they have their eyes closed"<br>"Billy looks too angry when Joe leaves"<br>"Tina looks a bit scary with her eyes closed while laughing"		Improved illustrations of facial expressions
More realistic illustrations:<br>"phone looks too big"<br>"Amy's room should be more messy"<br>"Billy needs more bruised and scratches for someone who's been in a car crash"		Reduced phone size<br>Added more mess to Amy's room<br>Added more scratches to Billy
Changes to core characters:<br>Joe: should look younger, softer facial expression and shorter, don't like hairband, change to beads<br>Tina and Billy: look like siblings, change Tina's hair colour to increase diversity as well<br>Emma: looks old and looks like a mum, change short haircut to a high ponytail to make her look younger		Joe: added beads to his hair<br>Tina changed from blonde to red hair<br>Emma changed from short bob to ponytail hairstyle
	Language	
We don't use the word yarn, more for adults	"kids say chat rather than yarn"	Taken out young characters saying 'yarn', only adult characters are using the word
"wouldn't say weed, probably say smoking"		In some instances changed "smoking weed" to "smoking something" or "smoking" (only when clear it is referring to weed)
"Doesn't sound good to have the characters say 'my mum says'"		Taken out the younger characters referring to adults when sharing key messages to further increase peer led nature of the program.

Table S2. Expert Advisory Group feedback and changes made to the program.

Expert feedback	Corresponding change to the program
	Content
Unsure about the inclusion of "notes specifically related to Aboriginal and Torres Strait Islander students", if including, they need to be included every lesson and they need to have a clear call to action for the teacher	- Included notes in every lesson<br>- Included call to action in each of the notes.
"Given that short and long-term effects [of cannabis] depend on method of administration, I was surprised this wasn't included somewhere."	- Included information about the way cannabis is administrated and the impact that this has on the effects of cannabis
Given that cultural elements were found as an important factor in effective drug prevention for Indigenous youth, it would be beneficial to have a cultural activity in each lesson	- Included a cultural activity in each lesson of the program.
	Technical
It is not intuitive on the website how you access the illustrated stories, change "Strong & Deadly Futures" to "cartoon"	Changed the name of the icon that leads to the illustrated stories
Font in the illustrations is quite small	Changed online display of the illustrations to increase slide and font size
Downloadable PDF with pages under each other makes it hard to navigate the PDF, recommend creating a booklet format	Created a booklet format for the PDFs of the teacher and student summaries and activities, including with a content page.
Difficulties with navigation	Technical updates were implemented to improve navigation.
Change menu button to be more in line with what is generally used as menu button	Suggestion implemented
	Language, illustrations etc
"Some terminology is quite middle class" and need more "Aboriginal English"	Changes made to language in the classroom activities and summary documents.
More images needed in the summaries	Suggestion implemented
Language needs to be simplified, including shorter sentences and dotpoints	Simplified language in the summaries and classroom activities documents, incorporating shorter sentences, more lists and dot points, and diagrams to explain concepts
Brainstorm questions need to be open ended questions	Suggestion implemented.
